# A Technology-Supported Guidance Model to Support the Development of Critical Thinking Among Undergraduate Nursing Students in Clinical Practice: Concurrent, Exploratory, Flexible, and Multimethod Feasibility Study

**DOI:** 10.2196/43300

**Published:** 2023-04-26

**Authors:** Jaroslav Zlamal, Edith Roth Gjevjon, Mariann Fossum, Simen A Steindal, Andréa Aparecida Gonçalves Nes

**Affiliations:** 1 Department of Bachelor Education in Nursing Lovisenberg Diaconal University College Oslo Norway; 2 Department of Health and Nursing Sciences University of Agder Kristiansand Norway; 3 Faculty of Health Studies VID Specialized University Oslo Norway; 4 Department of Postgraduate Studies Lovisenberg Diaconal University College Oslo Norway

**Keywords:** technology, guidance model, critical thinking, feasibility, nursing, nursing education, medical education, nursing student, digital intervention, mobile app, clinical practice

## Abstract

**Background:**

There is widespread recognition and acceptance of the need for critical thinking in nursing education, as it is necessary to provide high-quality nursing. The Technology-Supported Guidance Model (TSGM) intervention was conducted during clinical practice among undergraduate nursing students and aimed to support the development of critical thinking. A major element of this newly developed intervention is an app, Technology-Optimized Practice Process in Nursing (TOPP‑N), combined with the daily guidance of nursing students from nurse preceptors and summative assessments based on the Assessment of Clinical Education.

**Objective:**

The main objective of this study was to assess the feasibility of a newly developed intervention, TSGM, among undergraduate nursing students, nurse preceptors, and nurse educators. Further objectives were to assess the primary and secondary outcome measures, recruitment strategy, and data collection strategy and to identify the potential causes of dropout and barriers to participant recruitment, retention, intervention fidelity, and adherence to the intervention.

**Methods:**

This study was designed as a concurrent, exploratory, flexible, and multimethod feasibility study of the TSGM intervention that included quantitative and qualitative data from nursing students, nurse preceptors, and nurse educators. The primary outcome measures were the feasibility and acceptability of the intervention. The secondary outcomes included the suitability and acceptance of the outcome measures (critical thinking, self-efficacy, clinical learning environment, metacognition and self-regulation, technology acceptance, and competence of mentors); data collection strategy; recruitment strategy; challenges related to dropouts; and hindrances to recruitment, retention, and intervention fidelity and adherence.

**Results:**

Nursing students, nurse preceptors, and nurse educators had varied experiences with the TSGM intervention. We identified factors that make the intervention feasible and challenging and may influence the feasibility, acceptability, dropout rate, adherence, and fidelity of the intervention. We also identified areas for future improvement of the intervention.

**Conclusions:**

The use of a newly developed intervention, TSGM, is feasible and accepted by undergraduate nursing students, nurse preceptors, and nurse educators; however, refinement and improvement of the intervention and the TOPP‑N app, improvement in intervention management, and mitigation of negative factors are necessary before a randomized controlled trial can be performed.

**International Registered Report Identifier (IRRID):**

RR2-10.2196/31646

## Introduction

### Background

Critical thinking is widely recognized and well established in nursing education [1-3] because the ability to identify and assess nursing care requires critical thinking [4,5]. However, interventions to improve critical thinking in nursing education and ways to facilitate it among undergraduate nursing students are currently debated without definitive conclusions [6]. The most frequently emphasized critical thinking skills in higher education in European countries are analysis and evaluation skills. Immersion is a commonly used approach for supporting the development of critical thinking [7]. However, a mixed methods systematic review [8] revealed a lack of studies on how to facilitate the development of critical thinking using technological tools among nursing students in clinical settings.

According to several authors [6,9,10], it is essential to conduct studies using experimental designs, such as randomized controlled trials (RCTs), to determine the most effective strategy to foster critical thinking among nursing students. A feasibility study, pilot study, or both should be conducted before the execution of an RCT to strengthen the justification, design, and planning of the study [11].

In this paper, we present the results of a feasibility study conducted before a planned RCT to evaluate a newly developed intervention, the Technology-Supported Guidance Model (TSGM), aimed at supporting the development of nursing students’ critical thinking skills. An app, Technology-Optimized Practice Process in Nursing (TOPP‑N), was developed by the first and last authors (JZ and AAGN) in collaboration with nursing students, nurse preceptors, and nurse educators. During clinical practice, nursing students, nurse preceptors, and nurse educators used the app daily, thereby generating new insights among them. The aim of such use is to improve the clinical practice of nursing students in general. In addition, as part of the intervention, a summative assessment of the students’ progress in clinical practice was conducted using the Assessment of Clinical Education (AssCE) [12] (digitalized in the TOPP-N app), either in person or through Zoom meetings (Zoom Video Communications Inc) [13]. This intervention is based on metacognition [14] and constructive alignment [15] and consists of using the TOPP-N app in conjunction with daily guidance. Protocols for the feasibility study [16] and RCT [17] have been published previously.

### Aim

This study aimed to assess the feasibility of the TSGM intervention during clinical practice for undergraduate nursing students, nurse preceptors, and nurse educators before an RCT.

### Objectives

The study objectives were to evaluate the feasibility and acceptability of the newly developed TSGM intervention in clinical practice among undergraduate nursing students, nurse preceptors, and nurse educators; to assess the feasibility and suitability of the primary and secondary outcome measures; to assess the recruitment strategy; to assess the data collection strategy; and to identify potential causes of dropout and barriers to participant recruitment, retention, intervention fidelity, and adherence to the intervention.

### Research Questions

The research questions were as follows:

1. How feasible and acceptable is the newly developed TSGM intervention among undergraduate nursing students, nurse preceptors, and nurse educators?
2. Are the outcome measures feasible and suitable for measuring the effect of the intervention?
3. How feasible is the chosen data collection strategy?
4. How suitable is the participant recruitment strategy?
5. What causes dropout and which hindrances can occur in relation to recruitment, retention, and intervention fidelity and adherence?
6. How can these hindrances be minimized?

## Methods

### Overview

We used a concurrent, exploratory, flexible, and multimethod design in this study. As a result of this design, it was possible to combine multiple research questions; a variety of research methods; and multiple ways to analyze data across different samples, settings, and times [18]. Furthermore, during the feasibility study, the chosen design allowed us to make necessary changes that could improve the design and execution of the final RCT [19]. The study was written according to the SPIRIT (Standard Protocol Item: Recommendations for Interventional Trials) checklist [20], Medical Research Council Framework for Complex Interventions [11], and TIDieR (Template for Intervention Description and Replication) [21]. Deviations from a previously published study protocol [16] are summarized in Multimedia Appendix 1 [22].

### Sampling, Participant Characteristics, and Recruitment

A nonprobability convenience sampling strategy was used to recruit nurse preceptors, nurse educators, and undergraduate nursing students. The eligibility criteria are summarized in Textbox 1.

All nursing students, nurse preceptors, and nurse educators who participated in clinical practice or guidance in nursing homes that cooperated with the project were invited to participate in this feasibility research study.

Recruitment was performed by the first author (JZ) and the last author (AAGN). Owing to the COVID-19 pandemic and physical meeting restrictions, web-based Zoom meetings [13] were held from January 10, 2021, to February 14, 2021, to recruit participants. During these Zoom meetings [13], detailed information was provided about the feasibility study, and the participants’ questions were answered. The meetings were supplemented by written information published on the Canvas Learning Management Platform (Instructure Inc) [23]. Although the sampling was performed at the individual level, we had to consider where the students had their clinical practice. A feasibility study in the same nursing home, in which some students were involved in the intervention and others were not, would present a challenging and disturbing situation for all. Consequently, the Lovisenberg Diaconal University College (LDUC) decided that all students who had clinical practice in the nursing home where the intervention was being conducted had to follow the intervention. Before the start of the feasibility study, the students were given the option of staying at a nursing home where the intervention was conducted or being relocated to another clinical practice site where no intervention took place. At the same time, to ensure voluntary participation in the research, the students were given the option to participate or opt out of participation in research activities. We defined *participants* as those who had signed an informed consent form and maintained it, regardless of their degree of following the intervention or participating in research activities.

We defined *nonparticipants* as “users” and those who had followed the intervention but did not sign an informed consent form. The term *research activity* refers to the completion of measurement instruments with or without participation in focus group interviews. Similarly, a decision was made with the cooperation of nursing homes at the University of Agder (UoA). Nurse preceptors and nurse educators also had the option of declining to guide students in the nursing homes where the intervention was being conducted.

**Eligibility criteria.**

- Nursing students
  First-year undergraduate nursing students at Lovisenberg Diaconal University College (LDUC) or University of Agder (UoA)
- Nurse preceptors
  Registered nurses working in nursing homes in participating institutions and guiding students in clinical practice
- Nurse educators
  Nurse educators working at LDUC or UoA and guiding students in clinical practice

### Study Setting

In total, there were 4 nursing homes in the County of Oslo (n=1, 25%) and the County of Agder (n=3, 75%).

### Description of the TSGM Intervention and Activities

Nursing students, nurse preceptors, and nurse educators used the TOPP-N app. The TOPP-N app has 2 modules: a guidance module and an assessment module. The guidance module is recommended to be used daily but can be tailored to user needs, whereas the assessment module is used for summative assessment during mid- and final-term clinical practice assessment. Nursing students were required to complete e-reports in the guidance module before and after their shifts in clinical practice. In these e-reports, nursing students planned their learning activities and chose from learning points presented by the TOPP-N app, which constitutes a checklist built on AssCE [12]. In e-reports, nursing students also indicated whether they needed assistance with specific learning activities on a scale based on AssCE [12]. Students could also provide further written elaboration on their learning needs.

Nurse preceptors were responsible for providing feedback and guidance to students on their learning needs based on daily e-reports, which were provided in the TOPP-N app by directly writing or recording voice feedback. Both nursing students and nurse preceptors also needed to fill out a “need for guidance scale,” which is a 5-point Likert scale of perceived need for guidance and ranges from “very little extent” to “very large extent.” In addition, nurse preceptors provided face-to-face guidance. This daily interaction between nursing students and preceptors in the TOPP-N app generated an overview that was accessible to all parties. The role of nurse educators was to monitor the progress of nursing students. To help nurse educators monitor student progress efficiently, automatically generated notifications were embedded in the TOPP-N. When the TOPP-N app detected a discrepancy between a student’s learning needs and a nurse preceptor’s judgment of these needs in 4 consecutive days, a notification was sent to the nurse educator, who could then review the discrepancy and intervene, if necessary. Nursing students, nurse preceptors, and nurse educators could also directly communicate through messages in the TOPP-N app, which also includes a separate assessment module built on AssCE [12].

The intervention lasted 6 weeks at LDUC and 8 weeks at UoA, as a result of varied COVID-19–related measures in different parts of Norway. Normally, the clinical practice period in Oslo, where the LDUC is situated, is 8 weeks. Owing to the COVID-19 pandemic, the period was reduced to 6 weeks on request from and in collaboration with the County of Oslo.

In this feasibility study, the intervention was supervised by the first author (JZ) and the last author (AAGN). Furthermore, a “superuser” was engaged; the superuser was a student who had extensive experience with the TOPP-N app through earlier participation in the design and development of the intervention.

**Figure 1:**
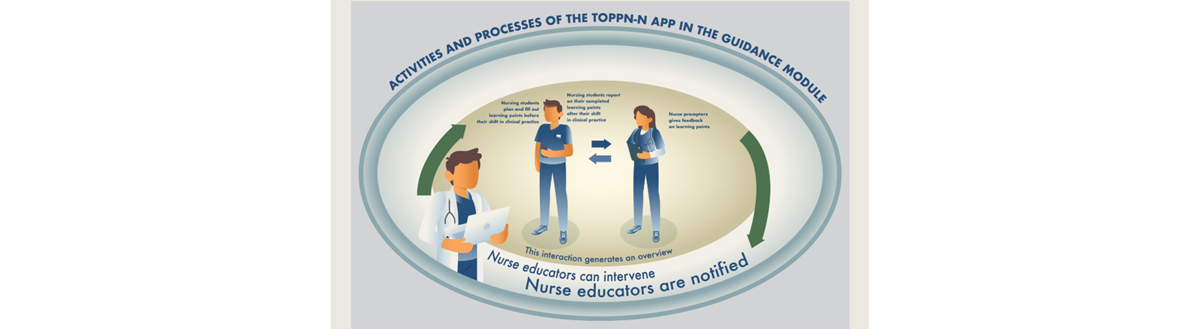
The activities and processes of the Technology-Optimized Practice Process in Nursing (TOPP-N) app in the guidance module.

To ensure effective communication with the participants and provide the necessary support and updates regarding the feasibility study, we established an “invitation only” Facebook group. As part of the feasibility study, we used Facebook to maintain a sense of motivation for participation. This Facebook group was run by the first author (JZ), the last author (AAGN), and the superuser. MOSO was responsible for technical development, maintenance, and support of the TOPP-N app. The activities and processes of the TOPP-N app in the guidance module are summarized in Figure 1 and have been previously published in a study protocol [16].

It is important to note that the approach to the TSGM intervention, especially the use of the TOPP-N app and guidance module, was different between the 2 educational institutions that participated in the project. At the LDUC, the use of the TOPP-N app was mandatory; nursing students were instructed to complete reports daily, and nurse preceptors were instructed to give students daily feedback. At UoA, although the use of the app was mandatory, the nursing students were encouraged to complete the reports as often as possible and at least once a week, and the nurse preceptors were encouraged to give the students feedback when the report was completed.

### Outcomes

The primary outcomes of this study were the feasibility and acceptability of the intervention. The secondary outcomes were the suitability and acceptability of outcome measures (critical thinking, self-efficacy, clinical learning environment, metacognition and self-regulation, technology acceptance, and competence of mentors); data collection strategy; recruitment strategy; challenges related to dropouts; and hindrance to recruitment, retention, intervention fidelity, and adherence.

### Sample and Sample Size

Our sample comprised undergraduate nursing students, nurse preceptors, and nurse educators. Following Billingham et al [24], we estimated the necessary total sample size for all participants to be 12 to 50.

### Data Collection

Both quantitative and qualitative data were collected through a plan for the in-person collection of quantitative data in the study settings. The quantitative data included TOPP-N app use data and responses to the questionnaire evaluating the feasibility study. Quantitative data were collected from a questionnaire administered on the web using Questback’s management system (Questback Group AS) [25] and from anonymous use data obtained via the TOPP-N app.

We also tested the following measuring instruments intended to collect information on the effectiveness of the intervention in the final RCT: Health Sciences Reasoning Test (HSRT), Self-Efficacy in Clinical Performance, Clinical Learning Environment, Supervision and Nurse Teacher, Technology Acceptance Model 3, Mentors Competence Instrument, and Self-Regulation and Metacognition in Clinical Practice. The HSRT instrument was administered through the Insight Assessment Testing interface, whereas the rest of the instruments were administered through Questback’s management system [25]. A summary of the characteristics of these instruments is provided in Multimedia Appendix 2 [26-31] and has been previously published in the study protocol [16].

The participants were asked to fill out one or several measuring instruments simultaneously. The total amount of time (before and after the intervention) expected to be used by participants on measuring instruments was 280 minutes for nursing students and 40 minutes for nurse preceptors and nurse educators.

The qualitative data included 1 focus group interview with each group of participants in April 2021 under the guidance of a researcher who acted as a moderator and another who acted as an assistant moderator. The researchers did not participate in the development or testing of this intervention. The interviews lasted 60 minutes, and they were conducted through the Zoom app owing to COVID-19–related restrictions and were recorded. On completion of the interviews, only voice recordings were retained for verbatim transcription, whereas video recordings were discarded. Qualitative data also included anecdotal feedback shared by participants in the dedicated Facebook group and via email. In addition, we collected data on sample demographics, such as the age of the participants, last completed education, and previous health care experience.

### Data Analysis

We analyzed quantitative data using DATAtab statistical analytical software (DATAtab Team) [32] and descriptive statistics, such as means, averages, differences, range, and percentages.

The focus group interviews were transcribed by an independent transcription company. The first author (JZ) machine translated the transcribed focus group interviews from Norwegian into English using the MateTranslate translation tool (Gikken) [33]. The translated text was machine translated back and forward to identify discrepancies. Discrepancies were manually corrected. Qualitative data were analyzed using semantic network analysis [34] and thematic analysis [35], using analytical and descriptive themes [36].

**Figure 2:**
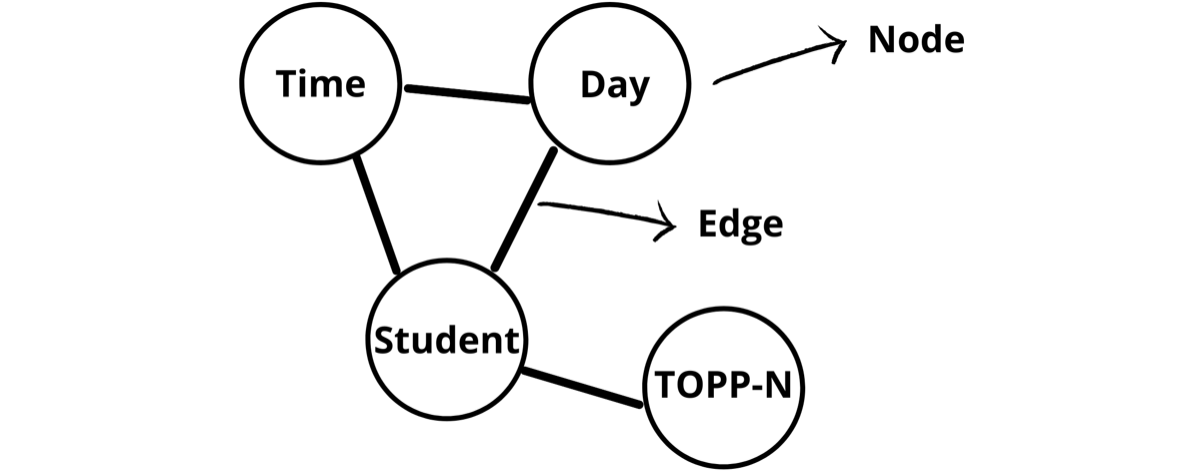
An example of nodes and edges from the transcribed text of focus group interviews. Adapted from Telatnik [38] and sourced by the authors. TOPP-N: Technology-Optimized Practice Process in Nursing.

The semantic network analysis draws on the principles of network analysis. The core principle of the semantic network analysis is to create a visual network from unstructured text [34] and subsequently explore various interactions, called *edges*, between nodes [37]. Nodes may represent various concepts or words [34]. In this study, the nodes are words in the text of a transcribed focus group interview (Figure 2 [38]).

We also refer to *degree centrality*, which represents the number of connections a node has with other nodes in a network [37]. Network analysis was conducted using the InfraNodus text network analysis tool (Nodus Labs) [39]. We used both quantitative and qualitative interpretations of the semantic network, depending on which approach was suitable [34].

We integrated the semantic network analysis as the first step of thematic analysis to get acquainted with and understand the initial meaning of the data from each focus group interview. Next, using the InfraNodus text network analytical tool (Nodus Labs) [39], the first author (JZ) created semantic network maps of the transcribed focus group interviews, with 1 semantic network map for each group: nursing students, nurse preceptors, and nurse educators. The semantic network files are provided in Multimedia Appendix 3. During this process, the first author (JZ) focused on nodes, edges, and degree centrality to qualitatively (visually) explore the semantic network and identify the prominent nodes. Thereafter, the first author uploaded the transcribed interviews to the MAXQDA tool (Verbi GmbH) [40]. On the basis of the results of the network analysis, the first author (JZ) identified each prominent node and adjacent text and coded the text using an initial code. After all nodes were identified in the text, the remainder of the text was coded according to the thematic analysis.

Text segments were coded according to their meaning units. The codes were organized in a codebook with an individual description of each code. After this initial coding process, units with the same meaning were independently coded by the last author (AAGN) according to the codebook. Then, we jointly looked at discrepancies among the codes and further collapsed, split, and reduced codes and edited code definitions to create descriptive themes.

This process was repeated until discrepancies and misunderstandings in code definitions were eliminated. After this process, we performed a final coding and calculated the intercoder reliability. Cohen κ was 0.65 for individual codes, indicating substantial agreement (range 0.61-0.80) between the coders [41].

The first author then continued the thematic analysis to create analytical themes, with the aim of gaining a new understanding.

### Ethics Approval

The study was approved by the Norwegian Centre for Research Data (reference number: 338576), LDUC, UoA, and the participating nursing homes. Before the feasibility study was initiated, agreements were signed with participating nursing homes.

Informed consent (Multimedia Appendix 4) was obtained digitally through Questback’s management system [25] and by the first author (JZ) and the last author (AAGN). Signed informed consent forms and adjacent sociodemographic data, together with data from all questionnaires, were stored in the Questback Management System [25]. We used only the deidentified data. HSRT was conducted using the Insight Assessment Testing system [42]. Only anonymous results are stored in the Insight Assessment System. A backup of all data was stored on a Kingston Data Traveller 2000 USB stick with AES 256-bit encryption (Kingston Technology Europe Co LPP). Access to these data was provided only to the first author (JZ) and the last author (AAGN). None of the authors of this study participated in the formal teaching, guidance, or evaluation of any participant in this feasibility study. No compensation was given to the participants for their participation in the study.

## Results

### Overview

In the following sections, we present the sample characteristics, followed by dropout rates, sociodemographic data, quantitative data on the results of anonymous use data from the TOPP-N app, and the results of quantitative semantic network analysis. Furthermore, we present qualitative data from focus group interviews and anecdotal feedback from the participants. Owing to the low response rate (in total, 8/8, 100%, participants; nursing students: n=4, 50%; nurse preceptors: n=3, 38%; and nurse educators: n=1, 12%), we chose not to present data from the responses to the questionnaire that evaluated the experience of the feasibility study. However, for the purpose of transparency [43,44], an overview of the participants’ responses is provided in Multimedia Appendix 5.

### Sample and User Characteristics

We invited 63 nursing students, nurse preceptors, and nurse educators, of whom 24 signed the informed consent form (Table 1). None of the participants withdrew their signed consent.

**Table 1:** Invited individuals, sample, and user participation in the project.

	Nursing students, n (%)	Nurse preceptors, n (%)	Nurse educators, n (%)
Used the TOPP-N^a^ app (invited; N=63)	32 (51)	27 (43)	4 (6)
Signed the informed consent (sample; n=24)	15 (62)	5 (21)	4 (17)
Completed at least 1 questionnaire (n=21)	15 (71)	2 (10)	4 (19)
Participated in the focus group interview (n=14)	5 (36)	5 (36)	4 (28)

^a^TOPP-N: Technology-Optimized Practice Process in Nursing.

### Dropout Rates

On the basis of a comparison of the response rate with the measuring instruments at the beginning of the intervention and after the completion of the intervention, we calculated the dropout rate, which varied between 42% and 75% between all groups. The summarized response rates among nursing students, nurse preceptors, and nurse educators, along with the dropout rates on these instruments, are provided in Table 2.

**Table 2:** Response rate and dropout rate according to the measuring instruments (N=63).

Measuring instrument	Participants answering at the start of the intervention, range^a^				Participants answering after the completion of the intervention, range				Dropout rate, n (%)
	NSs^b^	NPs^c^	NEs^d^	NSs		NPs	NEs		
HSRT^e^	11	N/A^f^	N/A	5		N/A	N/A	6 (54)	
SECP^g^	14	N/A	N/A	8		N/A	N/A	6 (42)	
CLES+T2^h^	—^i^	N/A	N/A	6		N/A	N/A	N/A	
TAM3^j^	12	—	4	5		2	1	For NS: 7 (58); for NP: N/A; for NE: 3 (75)	
MCI^k^	N/A	1	N/A	N/A		2	N/A	N/A	
SMCP^l^	4	N/A	N/A	5		N/A	N/A	N/A	
Evaluation of the feasibility study	—	—	—	4		3	1	N/A	

^a^For example, participants answering at the start of the intervention: n=0-14 (0%-100%). Participants answering after the completion of the intervention: n=0-8 (0%-100%).

^b^NSs: nursing students.

^c^NPs: nurse preceptors.

^d^NEs: nurse educators.

^e^HSRT: Health Sciences Reasoning Test.

^f^N/A: not applicable.

^g^SCP: Self-Efficacy in Clinical Performance.

^h^CLES+T2: Clinical Learning Environment, Supervision, and Nurse Teacher.

^i^Not answered.

^j^TAM-3: Technology Acceptance Model 3.

^k^MCI: Mentors Competence Instrument.

^l^SMCP: Self-Regulation and Metacognition in Clinical Practice.

### Nursing Students’ Sociodemographic Data

The sociodemographic data showed that among the participants who completed the sociodemographic questionnaire (n=13), the majority were women (n=10, 77%). For most participants (n=9, 69%), the nursing education they had started was their first higher education degree; 1 (8%) participant had a previous bachelor’s degree, and 3 (23%) did not answer this question. More than half of the participants (n=7, 54%) had long-term or previous work experience in health care settings.

### Results From the Quantitative Data

The use of TOPP-N made it possible to assess the range of app use for each user (Table 3). These data were provided to the research team by MOSO. The data were anonymized by an independent researcher. Use data from nurse educators were not available.

The results of the semantic network analysis are summarized in Table 4 according to the degree centrality [37].

The nodes “time” and “day” refer to the how often TOPP-N app was used, and the results indicate that nursing students and nurse preceptors were highly preoccupied by the amount of time spent on the TOPP-N app, whereas nurse educators were more occupied with the guidance procedures, which is reflected in the nodes “student” and “supervisor.” In addition, nurse educators were focused on procedures related to mid- and final-term assessments in clinical practice, which is reflected in the node “assessment.”

**Table 3:** Use data from Technology-Optimized Practice Process in Nursing (TOPP-N) users.

	Daily use of the TOPP-N app							
	Planning and reporting				Feedback			
	NS^a^				NPs^b^			
	LDUC^c^ (n=16)		UoA^d^ (n=16)		LDUC (n=13)		UoA (n=14)	
	Range	Mean (SD)	Range	Mean (SD)	Range	Mean (SD)	Range	Mean (SD)
Week 1 of clinical practice	1-5 times	2.93 (11.14)	0 times	N/A	0-5 times	2.08 (1.75)^e^	0 times	N/A
Weeks 2-6 of clinical practice	2-31 times	17.5 (7.16)	0-13 times	4.4 (4.31)	0-15 times	4.26 (2.67)	0-6 times	0.41 (0.99)

^a^NSs: nursing students.

^b^NPs: nursing preceptors.

^c^LDUC: Lovisenberg Diaconal University College.

^d^UoA: University of Agder.

^e^Three nurse preceptors did not use the TOPP-N app in week 1.

**Table 4:** Summary of the most prominent nodes according to their degree centrality.

Node	Degree centrality		
	Nursing students (n=2324)	Nurse preceptors (n=2390)	Nurse educators (n=3000)
Time	82	95	N/A^a^
Day	70	73	N/A
Student	N/A	N/A	100
Supervisor	N/A	N/A	79
Assessment	N/A	N/A	65

^a^N/A: not applicable.

### Results From the Qualitative Data

#### Results of Focus Group Interviews

Qualitative data were collected through focus group interviews with nursing students (focus group 1; 5/14, 36%), nurse preceptors (focus group 2; 5/14, 36%), and nurse educators (focus group 3; 4/14, 28%).

On the basis of the thematic analysis, 3 emergent themes were identified: facilitating factors that make the intervention feasible, challenges to the feasibility of the intervention, and needs for further development. Table 5 provides an overview of the analytical and descriptive themes, codes, and their descriptions.

**Table 5:** Overview of analytical themes, descriptive themes, codes, and their descriptions.

Analytical theme, descriptive theme, and code (identifier)			Description
**Facilitating factors that make the intervention feasible**			
	**Defined roles**		
		Nurse preceptors’ role (NURR)	Descriptions of the role of nurse preceptors in conjunction with the use of the app or guidance procedures; descriptions based on experiences or personal meaning
		Nurse educators’ role (NURE)	Descriptions of the role of nurse educators in conjunction with the use of the app or guidance procedures; descriptions based on experiences or personal meaning
		Students’ role (SR)	The role of students in the guidance process; describes expectations for the student role
	**User-friendliness of the app**		
		Advantages of using the app (AUP)	Description of advantages or positive effects when using the app during clinical practice; descriptions of inclusion or participation in development
		Motivation (MOT)	Motivation to use the app, follow guidance procedures, participate in research or for the development of the app or procedures; attitudes regarding the use of the TOPP-N^a^ app
**Challenges to the feasibility of the intervention**			
	**Time management**		
		Time (TIMAPP)	Users’ descriptions of the use of time in conjunction with the app or guidance procedures; descriptions of the workload associated with the use of the app and with following the guidance procedures
	**User limitations**		
		Misunderstanding (MISSUND)	Descriptions of various misunderstandings in relation to the use of the app or following the guidance procedures; used for misunderstandings expressed by nursing students, nurse preceptors, and nurse educators
		Stress or frustration related to the use of technological tools (ST)	Stress related to or in conjunction with the use of technological tools (not used for stress related to other factors)
		Research instruments (RI)	Descriptions of experiences related to filling out research questionnaires; descriptions of the characteristics of the questionnaires used in the research
	**App limitations**		
		Challenges to the use of the app (CHAPP)	Descriptions of pedagogical challenges or challenges to using the app; attitudes when using or not using the app (excluding management issues)
**Needs for further development to carry out the RCT^b^ study**			
	**Need for structure**		
		Support (SUPT)	Descriptions of support in conjunction with the use of the app (used for technical, pedagogical, or other support)
		Training (TR)	Description of experiences of or challenges to training before, during, or after using the app; descriptions of the need for information or expectations in relation to the use of the app
		Management anchoring (MAN)	Need for anchoring or foundation in management, either in health institutions or in educational institutions, in relation to the use of the app or participation in the intervention
	**Continuous app improvement**		
		App development (APPD)	Suggestions or ideas for future app development or changes to the app

^a^TOPP-N: Technology-Optimized Practice Process in Nursing.

^b^RCT: randomized controlled trial.

#### Facilitating Factors That Make the Intervention Feasible

We identified 2 facilitating factors that influenced the feasibility of the intervention: clearly defined roles and user-friendliness of the app. The clearly defined roles and expectations related to those roles were important to all participants. Nursing students emphasized that their role during the intervention was to learn. They experienced that they sometimes had to teach nurse preceptors how to use the TOPP-N app, which they perceived negatively as not being a part of their role as a student. Nurse educators emphasized their pedagogical role in the intervention and their role in ensuring that nursing students and nurse preceptors followed the intervention and adjacent tasks. One of the nurse educators (NE1) expressed, “In a way, you have to be a driving force for all parties to do their part.” The nurse preceptors described their roles in the intervention as tasks that were given and had to be completed.

Another facilitating factor was the user-friendliness and advantages of the TOPP-N app. Nursing students highlighted user-friendliness, but not nurse preceptors or nurse educators. Nursing students emphasized the simplicity and ease of use of the app. Among the advantages of using TOPP-N, all parties highlighted the ability to obtain an overview of the students’ clinical practice, the students’ progression toward learning goals, and the guidance that supervisors provided. One of the nurse preceptors (NP1) elaborated, “Yes, I also think, like the others, that the students may receive closer follow-up, and the work of the supervisors is made visible.”

In addition to the advantage of getting an overview, all the participants agreed that the TOPP-N app was, to a great extent, beneficial in mid- and final-term evaluations in terms of ease of use, accessibility, and the flexibility of the digital format of the AssCE [12].

Among facilitators of the intervention, motivation or being motivated was also identified as a factor that nurse educators (but not nurse preceptors or nursing students) were preoccupied with when using the TOPP-N app. The fact that nurse educators were part of the intervention was a motivating factor. The TOPP-N app was accessed and used mostly on mobile phones; however, nurse educators often used PC to a greater extent to access the TOPP-N app.

#### Factors That Make the Feasibility of the Intervention Challenging

The TOPP-N app use time was one of the topics that nursing students and nurse preceptors focused on the most, and they expressed that the use of TOPP-N was more time consuming than they had expected before the intervention. While nursing students and preceptors were preoccupied with the time aspect of daily use of the TOPP-N app, nurse educators focused more on app use time in mid- and final-term evaluations. A nursing student said:

But it’s, as also said here, that it takes a lot of time like that outside the actual practice period, so you spend a lot of time when all the points are to be filled in.

NS1

A nurse preceptor expressed her concerns about the app use time in the following way:

Yes. The system that we have started to use is quite usable, and it is quite good that you can give feedback on everyday life that goes along with the practice of students. But it takes time.

NP4

However, some nurse preceptors (NP3 and NP4) stated that the TOPP-N app use time was down to 5 to 10 minutes on daily e-reports. Nursing students tried to manage their time using the TOPP-N app to plan their shifts in clinical practice during their commute to clinical practice sites.

We identified several misunderstandings related to the use of the TOPP-N app, mainly among nursing students and preceptors. Some nursing students had the misconception that they had to have their phones with them the whole time to use the TOPP-N app:

I think TOPP-N takes away the time from patient contact...I probably felt that a little too much time was spent on documentation that didn’t directly benefit the patients. I felt a little guilt...having a private mobile in the uniform.

NS2 and NS4

The nursing students also believed that the TOPP-N app would interfere with patient contact and that what was planned in the learning points in the app at the beginning of a daily shift had to be the learning points that were followed during that day. One of the nursing students said:

It becomes something that takes time from patient contact, and the day in clinical practice was so unpredictable, even the supervisor didn’t know what we’re going to do that day, whether we’re allowed to catheterize or do wound care; we didn’t know.

NS3

We identified other misunderstandings among the nursing students who believed that when planning or evaluating their day of clinical practice in the TOPP-N app, they had to choose from all the available learning points presented by the app.

In addition, the nurse preceptors believed that the feedback the students received had to be given at the end of the day and that feedback during the day could not be given. One of the nurse preceptors said:

Because we take it verbally throughout the day. So, if I were to write on the TOPP-N app what I thought about the student, how she had done it during the day, she would not have heard it until after she finished work. That way, I think it is much better to provide it during the day. We did not identify any misunderstandings among nurse educators.

NP1

Frustration related to the use of technological tools was another factor that could make the feasibility of the intervention challenging. There were already many different technological tools that nursing students were using in clinical practice, and the addition of TOPP-N caused stress. A nursing student said:

Because, in addition to this app, we had a work mobile with its own app and documentation system, which was connected to the stationary documentation system, so you are supposed to go through all the different things then. So, it becomes a little stressful when you have many platforms in the digital world, and then you also have a lot of subjective realities to relate to in colleagues and patients.

NS2

Nurse preceptors and educators did not report any stress or frustration with the use of technological tools.

We also identified negative attitudes among nurse preceptors. According to nursing students, nurse preceptors expressed that it was not necessary to use the TOPP-N app every day or follow it strictly. The nursing students expressed that a demotivational factor in the intervention was when they actually used the TOPP-N app and completed e-reports at the start and end of their shift but never or only occasionally received feedback from nurse preceptors.

The participants said that participation in research activities and the completion of measuring instruments were time consuming; however, shorter instruments were easier to complete. All participants expressed that although the web-based measuring instruments were easy to access and use, the information in emails that followed the instruments was sometimes unclear and that emails were lost in spam folders or not received. The participants also reported technical issues when trying to access the web-based instruments.

During the course of the intervention, all participants experienced some degree of challenge with the TOPP-N app. For example, some nursing students found that filling out the learning points in the app was challenging and that it was not easy to understand what should be filled out and when. Nurse preceptors also experienced challenges in learning points, which, in their opinion, were not easy to distinguish as requirements at different levels of nursing education. All nurse educators highlighted the challenge of getting supervisors to write feedback regularly on student reports in the TOPP-N app.

#### Needs for Further Development

We identified themes related to the need for structure in relation to the TOPP-N app and the need for continuous improvement and development. The need for structure represents the need for support, training, and management anchoring of the TSGM intervention at both clinical practice sites and educational institutions. Nursing students provided feedback on the importance of training for those who used the TOPP-N app, especially when the supervisor was replaced. Nurse preceptors provided feedback on technical challenges, including problems downloading the app, logging into the app as a new user, and providing daily e-reports. One of the nurse preceptors wished for better training at the beginning of the intervention:

When I was about to download it, I was frustrated. It took some time. It was difficult to find all passwords. However, in the end, it worked. Therefore, perhaps we have a better recipe on how to download it? Because it was a bit time-consuming.

NP1

Nursing students praised the functionality of the Facebook support group. One of the nursing students explained:

And then we’re added to a Facebook group like that, so, if there was anything along the way, we could just ask questions there, and then we received answers quite frequently.

NS1

One of the nurse educators pointed out the importance of the availability of the superuser as part of the support for using the TOPP-N app:

Then it’s incredibly important that [the] superuser is easily accessible, that you get quick help, and that things are as intuitive as possible.

NE1

Anchoring the TSGM intervention and the use of TOPP-N in the management of both educational and health care institutions were considered important factors for the success of the intervention by nurse preceptors and nurse educators. Furthermore, nurse preceptors highlighted that not everyone had received adequate information before the intervention.

Some nurse educators pointed out the importance of nurse preceptors having sufficient resources when guiding nursing students:

So, it must also be organized so that [the] supervisors have time to familiarize themselves with that app and [have] time set aside for them to supervise and document and that it becomes routine in the department, that it becomes as [much] an everyday task as writing a report on patients.

NE1, NE2, and NE4

Nursing students and preceptors made suggestions for the future development of the TOPP-N app. The nursing students and nurse preceptors suggested that once-weekly use of the TOPP-N app for planning and then reviewing what had been done and completed, and the given feedback would be sufficient.

In the feasibility study protocol [16], we outlined the criteria for modifying or discontinuing the intervention. During the intervention, we did not identify any events that would require immediate termination of the intervention; however, we identified challenges and events that either required immediate adjustment of the intervention or would require its adjustment for future RCT. The challenges or events defined in our study protocol [16] were “amber progression criteria.” A summary of the challenges or events with suggestions for improvement is provided in Table 6.

**Table 6:** Summary of challenges during the intervention and suggestions for improvement.

Type of challenge or event	Description	Suggestion for improvement
Technological challenges during the training period and intervention	Problems with logging into the app; problems with proper operation of certain functions of the app, such as daily reports and feedback	Testing of the app before the beginning of the training period or at the start of the intervention; rapid resolution of technical challenges during the intervention
Lack of understanding of how the intervention and the TOPP-N^a^ app work	Some nursing students and nurse preceptors lacked understanding of the intervention and how to use the TOPP-N app	Providing repeated training, repeated information, and access to web-based training resources; establishment of a Facebook group or such for providing the necessary information before and during the intervention
Misunderstandings and negative attitudes	Nursing students and nurse preceptors misunderstood how the intervention worked and how the TOPP-N app should be used	Rapid clarification of misunderstandings by means of information through various channels: emails, learning platforms, Facebook group, or direct contact with participants; focus on mitigating negative attitudes
Challenges with recruitment and information flow	Challenges of recruiting nursing students and nurse preceptors via the internet and of communicating the necessary information on the web	Minimizing web-based recruitment and facilitating offline face-to-face recruitment; monetary incentives; allocation of time for participation
Challenges related to data collection	Web-based data collection was challenging and somewhat confusing owing to technical difficulties	Facilitating web-based data collection but in an offline face-to-face setting where participants can pose questions and get rapid answers and where technical issues can be promptly resolved; allocation of time to fill out questionnaires
Need for intensive supervision and oversight of the intervention	Labor-intensive supervision and oversight of the intervention; participant follow-up that was conducted by the first author (JZ), the last author (AAGN), and the superuser	Supervision is carried out by dedicated, full-time intervention supervisors: 1 supervisor for nursing students, 1 supervisor for nurse preceptors, and 1 supervisor for nurse educators

^a^TOPP-N: Technology-Optimized Practice Process in Nursing.

#### Experiences From the Course of the Intervention and Summary of Anecdotal Data

During recruitment, some nurse preceptors initially did not wish to receive information about the study, the TSGM intervention, or the TOPP-N app. Technical challenges occurred during the training period, which affected the training of the participating nursing students and preceptors. In the Facebook group, feedback during week 1 of the intervention was mostly about technical issues related to log-in and how to practically use the TOPP-N app.

In week 2, most of the participating nursing students and nurse preceptors used the TOPP-N app and had no further technical issues. The Facebook group associated with the participants from LDUC was used much more frequently than that associated with the participants from UoA. As students were allowed to comment on Facebook groups, 2 commented on their experiences using the TOPP-N app.

One student expressed content with the use of the app and perceived it as easy to use, whereas the other student expressed unhappiness with the use of the app and perceived it as cumbersome and unnecessary.

## Discussion

### Principal Findings

This study aimed to assess the feasibility of the TSGM intervention during clinical practice for undergraduate nursing students, nurse preceptors, and nurse educators before an RCT. Our findings suggest that the intervention is feasible and was accepted among undergraduate nursing students, nurse preceptors, and nurse educators. The successful completion and feasibility of the intervention appear to be dependent on several factors, such as clearly defined roles, sufficient training, and support, including technological support.

### Feasibility and Acceptability of the Intervention and Outcome Measures

Although the TSGM intervention and TOPP-N app use time was a recurring topic among the participants, the TOPP-N app use data showed that the TOPP-N app was used continuously throughout the clinical practice period, regardless of any individual’s participation in the intervention. The use data include both modules of the TOPP-N app (guidance and assessment) and show an increase in use from week 2 to week 6 of the intervention, especially among nursing students: 2 to 31 times (mean 17.5, SD 7.16) at LDUC and 0 to 13 times (mean 4.4, SD 4.31) at UoA. The difference in the range of use of the TOPP-N app can most probably be explained by the different lengths of intervention and approaches to the intervention between the 2 institutions. This was caused by the COVID-19–related measures that varied between the counties of Oslo (LDUC) and Agder (UoA).

One of the keys to the feasibility and acceptability of the intervention among the participants was the clearly defined role of nursing students, nurse preceptors, and nurse educators.

The students’ experience of being “a teacher” (teaching nurse preceptors how to use the TOPP-N app) might directly translate into the need for support and training given to nurse preceptors before the intervention and use of the TOPP-N app. This was especially true when a change in nurse preceptors occurred, and the nursing students experienced that the new nurse preceptor was not prepared to use the TOPP-N app. Although training and support were provided before and during the intervention along with intensive supervision by the first author (JZ) and the last author (AAGN) and the superuser, a change in nurse preceptors and, thereafter, a lack of information flow to those responsible for the intervention may have resulted in some nurse preceptors not being prepared for the TOPP-N. Technical challenges during the training period, that is, before the start of the intervention, may also have been a contributing factor. Clearly defined roles ensure group dynamics and determine how well a group works together [45,46], which in turn may also influence the feasibility and acceptability of this intervention.

A mismatch between the anticipated and received roles may cause role ambiguity: an unclear definition and understanding of roles and related tasks [45]. However, nurse preceptors’ perception of their role in guiding nursing students, either as an integral part of being a nurse or as an additional burden, could affect the actual role of preceptorship and the learning environment for nursing students [47]. The interview data and anecdotal feedback suggest that there is a need for an overall intervention facilitator who is near the clinical sites. In the case of the TSGM intervention, it was nurse educators who became the driving force for the intervention to work; however, this role might be understood as a task outside the nurse educators’ pedagogical scope.

The intended times for the use of the guidance module of the TOPP-N app were before the start of the day in clinical practice and then at the end. However, nursing students believed that they had to carry their phones all the time during the shift in clinical practice. We did not have data on how this misunderstanding had occurred. Before and during the intervention, there was much communication with the participants and a substantial amount of information sharing. Edwards et al [48] pointed out that misunderstanding is an integral part of communication and can easily occur.

Regarding the misunderstanding of choosing learning points in daily e-reports, these learning points were presented in a template based on AssCE [12] embedded in the assessment module and served as a guide to what nursing students could choose to focus on during their day in clinical practice. Nursing students also believed that their learning points could not be changed. A day in clinical practice is dynamic, and based on our program theory, as outlined in our study protocol [16], the planning of activities for the day was meant to help the students set learning goals and reflect.

Our findings suggest that the participants were concerned about the use time of the guidance module of the TOPP-N app, especially nursing students, which was surprising. In clinical practice, nursing students play a unique role as learners with the aim of learning, practicing skills, and socializing in the nursing profession [49]. This role should provide room, for example, to fill out e-reports in the TOPP-N as part of the learning process without feelings of stress owing to time pressure. Warne et al [50] pointed out that an important aspect of learning in the clinical setting is the pedagogical atmosphere, a positive environment that supports students’ learning. If this environment emphasizes “time and time usage,” students may experience the stress of time pressure when using the TOPP-N app. However, we also found contradictory findings related to app use time, which we attribute to the various misunderstandings that were identified in our data. Interestingly, one of the most praised features of TOPP-N among all the participants was the assessment module in mid- and final-term assessments; however, this feature was reported to be the most time consuming. It seems that daily reporting in the guidance module of the TOPP-N app is perceived as a greater burden in terms of stress experienced, especially by nursing students and preceptors. This may be due to a lack of information, a lack of understanding of how the intervention or TOPP-N app works, or negative attitudes. However, there were also positive experiences of perceiving the TOPP-N app as inspiring, exciting, and meaningful.

The participants suggested changing the intervention so that in the guidance module, students wrote e-reports and nurse preceptors gave feedback once a week. According to the program theory underpinning this study and previously published in the study protocol [16], the aim of the daily planning of learning goals, reporting of learning activities, and receiving feedback and guidance from nurse preceptors is to stimulate students to set goals, build learning strategies, and reflect on what is learned and needs to be further learned. Trying to achieve this aim by embedding weekly planning, reporting, and receiving feedback in the intervention seems counterproductive to the aim of establishing learning strategies and nurturing reflection. On the basis of this misunderstanding, the participants may have experienced various restrictions in their planning and execution of daily practice, which could lead to frustration and stress. In line with the suggestions of Edwards et al [48], the first author (JZ) and the last author (AAGN) attempted several times to correct misunderstandings by having open communication through Zoom meetings and sharing information through various channels, such as email or the Canvas learning management system. However, these misunderstandings persisted to some degree throughout the intervention period.

From the data, we identified that students experienced the introduction of TOPP-N as an additional technological tool on top of all the other technological tools that were already in use, which may have contributed to an additional burden. However, the TOPP-N app has become an important tool to ensure the follow-up of students and communication with the clinical field.

### Feasibility of the Data Collection Strategy

The data collection strategy aimed to ensure sufficient data collection and to provide participants with the information and support necessary to respond to the measurement instruments. The original plan for the data collection was to meet participants face to face, conduct quantitative data collection in person, and be available when participants filled out the measuring instruments. However, under COVID-19–related restrictions, this was not possible; therefore, we had to switch to a completely web-based data collection strategy. For focus group interviews, we planned Zoom meetings from the beginning to be flexible to conform to the different schedules of nursing students, nurse preceptors, and nurse educators. According to Kilinc and Firat [51], web-based data collection has many advantages, such as the freedom and flexibility of filling out research instruments at any time and place that are most suitable for research participants. However, one of the main disadvantages of web-based data collection is that participants have no option to ask questions or obtain instant answers or clarifications. We made a Facebook group available for questions and clarifications; however, we were not able to provide instant feedback, as we would in a face-to-face meeting, which may have influenced the data collection. Technological issues, such as difficulties in accessing questionnaires, may also contribute to the challenges in data collection [51].

### Recruitment Strategy

#### Overview

Owing to the COVID-19 pandemic restrictions, the recruitment of all participants was performed on the web, and we had already anticipated this when publishing the study protocol for this feasibility study. In the experiences of the first (JZ) and last (AAGN) authors, recruitment through Zoom app was challenging. The recruitment sessions were scheduled, for example, after students had attended their lecture; however, we encountered many students leaving the Zoom room on completion. Timing is essential when recruiting on the internet [52]. Recruitment before the start of the lectures may have been better suited to ensuring that as many potential participants as possible were present. We also tried to recruit students through email and announcements on Canvas with varying degrees of success. In comparison to our experience, Koo and Skinner [53] conducted web-based recruitment by email, sending 2109 emails to potential participants, of whom only 5 responded.

The recruitment of nurse preceptors was carried out by nurse managers at the given clinical site, which was a disadvantage, as the first author (JZ) and the last author (AAGN) had no direct contact with the participants during recruitment. Nurse educators were recruited directly from participating educational institutions. The incentive for participation in this study was that the students had the opportunity to learn about themselves, their own learning, and critical thinking and to contribute to improving nursing education.

For a participant to participate in a research study, there needs to be a sufficiently high level of incentive [53], and in this study, these incentives for participants may not have been motivating enough for them to complete the research activities.

#### Dropout, Retention, Adherence, and Intervention Fidelity

For nurse educators, personal motivation was an important facilitator for participating in the intervention. However, this was not the case for nursing students or nurse preceptors, and their motivation to participate in the intervention may have been affected by their experiences of stress. Motivation is an important factor, as it is directly related to how well a mobile app will be adopted by users [54], and it relates to the retention of the intervention. Duncan et al [55] pointed out that strategies to improve participant retention in an intervention often include monetary incentives or electronic prompts; however, we did not use any monetary incentives in this intervention. The established Facebook group also ensured intervention retention by engaging the participants and maintaining their interests.

The difference in motivation among nurse educators, nurse preceptors, and nursing students may be explained by nurse educators’ previous involvement in both the development and testing of the TOPP-N app and TSGM intervention over time. User involvement in app development can positively influence user satisfaction [56]. However, our data suggest that the attitudes of nurse preceptors may negatively affect nursing students’ perception of the TOPP-N app. In addition, the lack of feedback from nurse preceptors in the guidance module, despite the use of the TOPP-N app by nursing students, may have contributed to the lack of motivation. Such attitudes and experiences may threaten the fidelity of the intervention. The fidelity of a study’s intervention refers to the degree to which participants receive the proposed intervention or the instructions described in the study protocol [57].

Vallant and Neville [47] also pointed out that the attitudes of nurse preceptors may influence the learning of nursing students. Nursing students are dependent on nurse preceptors with respect to learning, and they may be inclined to agree with nurse preceptors to avoid repercussions. Thus, adherence to the intervention and intervention retention may be negatively influenced.

Another important factor that influences the future use of an app is its user-friendliness. Overall, the nursing students found the TOPP-N app easy to use and user-friendly; however, these findings were not echoed by nurse preceptors or nurse educators, although these groups pointed out the advantages and benefits of using the TOPP-N app, such as the overview of guidance and improved guidance. Previous research on motivation to continue using an app indicates that an app that is easy to use will have the highest chance of continuing to use [58].

The term *dropout* can be defined as the point at which outcome data are missing after a specified period [59]. The large dropout rate, ranging from 43% to 75% across all the participants in this study, was probably due to web-based recruitment and participation. Galesic [60] emphasized that web-based surveys are associated with a high dropout rate, affected by factors such as the subjective interest of the participants and the experienced burden. Although we do not have data that clearly suggest that the dropout rate was caused by the many measuring instruments that the participants were asked to complete, this may have been a contributing factor. However, other reasons for the high dropout rate may have been the COVID-19 pandemic and the need for participation at different time points.

All participants experienced challenges with the use of the TOPP-N app, mostly related to filling out daily reports in the guidance module and technological issues, which were quickly amended by the first author (JZ) and the last author (AAGN) and the superuser, who intensively supervised the intervention (more than initially expected), providing continuous support, information, and guidance. In addition, to ensure continuous use of the app and progress of the intervention, 2 more Zoom workshops, informational meetings, and informative comments in the Facebook group were organized during the course of the intervention. Despite the fact that information and training were provided before and during the intervention, intensive supervision was provided, and challenges may have occurred because of suboptimal information flow. Good information flow is a necessity for, for example, good working organization and functioning; to achieve this, information needs to be given effectively at the right time, and it needs to address the needs of those who are receiving the information [61]. We believe that this need for information and training is closely related to management anchoring, which all the participants believed was an important element of the intervention. In complex interventions, management plays a crucial role in supporting participants to ensure that an intervention succeeds [62].

### Strengths and Limitations

This feasibility study has several strengths. We conducted a flexible, multimethod feasibility study that included both quantitative and qualitative data. The thematic analysis with the semantic network analysis was used to strengthen the validity of the findings from the qualitative data. To establish the dependability of the thematic analysis, we conducted textual coding with 2 authors coding the same text and calculated the intercoder reliability using Cohen κ.

This study has several limitations. The decision to adopt a different intervention approach made by the educational institutions participating in this feasibility study, LDUC and UoA, after the study had started may have affected their participation in the TSGM intervention. Another limitation is the low response rate of the measuring instruments and the high dropout rates associated with these instruments. Consequently, we chose not to include the results of these questionnaires, which may have provided limited insight into the experiences of a wider group of participants than the focus group interviews could have provided.

The intervention also occurred during the peak of the COVID-19 pandemic, with rapidly changing restrictions, regulations, and information. The students were required to adjust to a completely digital learning environment. The duration of clinical practice was reduced from 8 to 6 weeks at LDUC while maintaining the same workload. The clinical practice was not reduced at UoA and was kept for 8 weeks. Contact with nurse educators was limited to communication via email or Zoom app; the same applies to the first author (JZ), the last author (AAGN), and the superuser who supervised the intervention. This, combined with the introduction of a completely new intervention with an app, may have caused cognitive overload.

### Conclusions

Despite the limitations of this feasibility study, we were able to confirm the feasibility of the TSGM intervention and identify important findings that will inform future RCT. The intervention is feasible during clinical practice among undergraduate nursing students, nurse preceptors, and nurse educators. However, close attention should be paid to positive factors that make the intervention feasible, such as clearly defined roles and the user-friendliness of the TOPP-N app, as well as to negative factors that make the intervention challenging, such as time management, user limitations, and app limitations. To mitigate the factors that can negatively affect training, sufficient information before and during the intervention and intensive supervision and management anchoring in both educational institutions and clinical sites should be the primary focus. Furthermore, the TOPP-N app should be continuously developed and improved in close cooperation with the users.

## Appendix

Multimedia Appendix 1 Deviations from the study protocol.

Multimedia Appendix 2 Overview of measuring instrument.

Multimedia Appendix 3 Semantic network map in .svg file format.

Multimedia Appendix 4 Informed consent—English version.

Multimedia Appendix 5 Overview of quantitative data not included in the study.

## Abbreviations

AssCE: Assessment of Clinical Education
HSRT: Health Sciences Reasoning Test
LDUC: Lovisenberg Diaconal University College
RCT: randomized controlled trial
SPIRIT: Standard Protocol Item: Recommendations for Interventional Trials
TIDieR: Template for Intervention Description and Replication
TOPP-N: Technology-Optimized Practice Process in Nursing
TSGM: Technology-Supported Guidance Model
UoA: University of Agder

## Data Availability

The data sets generated and analyzed during this study are available from the corresponding author on reasonable request.
