# Supplementary material for: A Technology-Supported Guidance Model to Support the Development of Critical Thinking Among Undergraduate Nursing Students in Clinical Practice: Concurrent, Exploratory, Flexible, and Multimethod Feasibility Study
Source: JMIR Form Res. 2023 Apr 26;7:e43300. doi: 10.2196/43300 (PMC10173047; doi:10.2196/43300)
Supplement: Multimedia Appendix 1 [file formative_v7i1e43300_app1.docx]

**Multimedia Appendix 1.** Deviations from the study protocol.

| Planned approach described in the protocol | Type, description, and justification of the deviation from the protocol |
| --- | --- |
| Design  Mixed-methods study design. | Type of deviation: clarification/change  After publication of the study protocol, we adjusted the wording and the actual design to a “multi-method feasibility study design” to better reflect the actual study design and subsequent methods. |
| Methods  We planned to use thematic analysis of qualitative data. | Type of deviation: addition  In addition to thematic analysis, we conducted a semantic network analysis that was combined with thematic analysis in order to strengthen our analysis and the findings from the qualitative data. |
| Methods  We planned for an integration of the quantitative and qualitative data through side-by-side comparison and interpretation in the Discussion section of the study. | Type of deviation: clarification/change  In line with our multi-method design, we present the results of semantic network analysis, thematic analysis, and quantitative data separately. These separate results have been discussed in the Discussion section of the study. |
| Aim of the study  The overall purpose of this study was to explore the feasibility of a Technology Supported Guidance Modell (TSGM) intervention for nursing students in clinical practice. | Type of deviation: clarification/change  We adjusted the aim to reflect that this study is conducted prior to a planned future randomized controlled trial (RCT).  The aim of this study was to assess the feasibility of the TSGM intervention during clinical practice for undergraduate nursing students prior to an RCT. |
| Sampling and sample size  We planned a consecutive sampling strategy.  We planned for a sample of 12 to 50 participants, specified as 32 nursing students (16 from LDUC and 16 from UoA) and 27 nurse preceptors (13 from LDUC and 14 from UoA). | Type of deviation: clarification/change  We changed our approach to convenience sampling for practical reasons: the availability of participants, the given time frame, accessibility, and willingness to participate.  We specified that we would focus on the total sample size and not on each group, which, according to our estimation, should be between 12 and 50 participants. |
| Statistical analysis  We aimed to calculate means, medians, standard deviations, skewness, and kurtosis. | Type of deviation: clarification/change  We calculated means, averages, differences, frequency, and percentages as this was most suitable for the purpose of the study after the change of design and methods. |
| Recruitment and data collection  We planned for face-to-face recruitment and data collection (for the measuring instruments) combined with online recruitment and online data collection (for the focus group interviews). | Type of deviation: clarification/change  As a consequence of COVID-19 restrictions, we had to switch to completely online recruitment and data collection. |
| Outcomes  Primary outcome: critical thinking. The secondary outcomes were self-efficacy, clinical learning environment, metacognition and self-regulation, technology acceptance, and the competence of mentors. | Type of deviation: clarification/change  We reframed and specified the primary and secondary outcomes in order to better reflect the aim of the feasibility study.  Primary outcome: the feasibility and acceptability of the intervention. Secondary outcomes: the suitability and acceptability of outcome measures (critical thinking, self-efficacy, clinical learning environment, metacognition and self-regulation, technology acceptance, and competence of mentors), data collection strategy, recruitment strategy, challenges to dropout, hindrances to recruitment, retention, and intervention fidelity and adherence. |
| Additional tool for analysis  InfraNodus Analytical Tool | Type of deviation: addition  In order to perform the semantic network analysis, we needed to use an analytical tool and chose the InfraNodus Analytical Tool for its ease of use and versatility. |
| County of Kristiansand | We corrected the name of the county to its currently correct version: the County of Agder. |

(Table adapted from Kinnear)
