# Supplementary material for: A Technology-Supported Guidance Model to Support the Development of Critical Thinking Among Undergraduate Nursing Students in Clinical Practice: Concurrent, Exploratory, Flexible, and Multimethod Feasibility Study
Source: JMIR Form Res. 2023 Apr 26;7:e43300. doi: 10.2196/43300 (PMC10173047; doi:10.2196/43300)
Supplement: Multimedia Appendix 2 [file formative_v7i1e43300_app2.docx]

**Multimedia Appendix 2.** Overview of measuring instrument.

| Measuring instrument | Characteristics of the instrument | Internal validity |
| --- | --- | --- |
| Health Sciences Reasoning test (HSRT) | Multiple choice test  38 questions  Measurement of overall level of critical thinking  Measurement of detailed scores of analysis, interpretation, inference, evaluation, explanation, induction, deduction and numeracy | Cronbach's alpha of 0.76 for the overall instrument [56] |
| Self–Efficacy in Clinical Performance (SECP) | Measurement of self–efficacy on 37 items in four subscales: assessment, diagnosis and planning, implementation and evaluation. | Cronbach's alpha for each item ranging from 0.90 to 0.92 [57] |
| Clinical Learning Environment, Supervision and Nurse Teacher (CLES+T2) | Measurement of satisfaction with the clinical learning environment on 45 items in three major themes: learning environment, supervisory relationship and role of the nurse teacher | Cronbach's alpha for each item ranging from 0.81 to 0.98 [58,59] |
| Technology Acceptance Model 3 (TAM 3)  Mentors Competence Instrument (MCI) | Measurement of acceptance of new technology on 37 items.  Self-assessment of own competence when mentoring nursing students in clinical settings. | Cronbach's alpha for each item ranging from 0.77 to 0.87 [60]  Cronbach's alpha values for the subscales observed ranged from 0.76 to 0.90. [61] |
| Self–Regulation and Metacognition in Clinical Practice (SMCP) | Measurement of level of use of self–regulation and metacognitive processes; measured on 11 items | Data not available |
| Sociodemographic data | Year of birth, gender, last completed education, length of employment in health care with direct patient contact | Data not available |
| Evaluation of the feasibility study | Evaluation of participation in the feasibility study | Data not available |

HSRT: Health Sciences Reasoning Test, SECP: Self-Efficacy in Clinical Performance, CLES+T2: Clinical Learning Environment, Supervision and Nurse Teacher, TAM3: Technology Acceptance Model 3, MCI: Mentors Competence Instrument, SMCP: Self–Regulation and Metacognition in Clinical Practice
