# Supplementary material for: A Technology-Supported Guidance Model to Support the Development of Critical Thinking Among Undergraduate Nursing Students in Clinical Practice: Concurrent, Exploratory, Flexible, and Multimethod Feasibility Study
Source: JMIR Form Res. 2023 Apr 26;7:e43300. doi: 10.2196/43300 (PMC10173047; doi:10.2196/43300)
Supplement: Multimedia Appendix 4 [file formative_v7i1e43300_app4.doc]

# Multimedia Appendix 4. Informed consent—English version.

# Do you want to participate in the research project Technology-supported guidance model to stimulate nursing students' critical thinking and competence

*(Technology-supported guidance model to stimulate nursing students’ critical thinking and competence)*

This is a question for you about participating in a research project. In this document, we provide you with information about the aims of the project and what participation will mean for you.

**Purpose**

The purpose of this research project is to test a newly developed guidance model in clinical practice and its effect on the development of critical thinking and nursing competence. The guidance model consists of a guidance application, digital practice assessment and digital meetings. The research project is linked to part of a doctoral thesis.

**Who is responsible for the research project?**

PhD candidate Jaroslav Zlamal, Lovisenberg Diaconal University College is responsible for the implementation of the project. Associate Professor Andrea Aparecida Goncalves Nes, Lovisenberg Diaconal University College is the Phd candidate`s supervisor, and is a collaborator in the project.

**Why are you being asked to participate?**

# In the project, we want to find out how the guidance model affects the development of critical thinking and nursing competence in nursing students who are in clinical practice. In addition, we want to investigate other factors that can influence the development of critical thinking and nursing competence, such as mastery beliefs, satisfaction with the clinical practice environment and guidance, as well as acceptance of the use of new technology. The project will be carried out among first-year students of the Bachelor in Nursing at Lovisenberg Diaconal University College. You are asked about participating in the project because you are a first-year student in the nursing program at Lovisenberg Diaconal University College. It is planned that up to 270 students will participate in the research project.

# What does participating mean for you?

The project is carried out in 2 phases.

Phase 1: a feasibility study (feasibility study) - up to 16 participants

Phase 2: a randomized controlled trial (RCT) – up to 250 participants with two groups. A control (which follows the usual guidance scheme at Lovinsenberg Diaconal University College) and intervention group (which follows the newly developed guidance model).

The intervention group will carry out their practice period according to the new guidance model, using a mobile application, digital practice assessment and digital meetings. The control group will carry out the practice period according to the ordinary guidance model (without the use of a mobile application, digital practice assessment).

Participation in the project means that you:

Conducts a practice period in accordance with the new guidance model or according to the ordinary guidance model

You fill in a set of questionnaires, see table 1 (Norwegian version of the questionnaires)

Enrolled participants will be randomly assigned whether they will participate in the feasibility study or randomized controlled trial (RCT). If you participate in the feasibility study, you cannot participate in a randomized controlled trial (RCT).

If you are participating in a randomized controlled trial (RCT), you will be assigned to either a control or intervention group.

If you participate in the feasibility study, you will be asked after the conclusion whether you want to participate in a focus group interview. A focus group interview is a form of group interview where other participants and researchers meet and discuss, gain experience with the use of the newly developed guidance model and experiences from participation in the feasibility study. The focus group interview will last approx. an hour and each group will consist of approx. 10 participants (approx. 8 students and 2 researchers who will lead the focus group interview). The focus group interviews will be audio recorded. The participants' names will not be given during the interview.

Table 1: Overview of the questionnaire to be filled in when participating in the project

| Name of questionnaire/test | Your contribution | When? |
| --- | --- | --- |
| Registration for the project. | You register for the project using a registration form created in Questback. You are asked to provide your name, email address, gender, age, previous education and previous experience from the healthcare system. | When registering for the project. |
| The Norwegian version of the Health Sciences Reasoning Test (HSRT). | This test measures the level of critical thinking. In this electronic questionnaire, you are asked to answer various questions/tasks. Your level of critical thinking will be calculated based on how you answer the various tasks given in the test.You will receive the form by email. It takes approx. 50 minutes to answer the questionnaire. | Before the start of the clinical practice period.  After completing the clinical practice period.  3 and 9 months after the last day you completed your clinical practice. |
| The Norwegian version of the Nurse Competence Scale (NCS) | This electronic questionnaire measures the level of your nursing competence. This is done by making a self-assessment of various claims related to nursing competence. It takes approx. 30 minutes to fill in the questionnaire. | Before the start of the clinical practice period.  After completing the clinical practice period.  3 and 9 months after the last day you completed your clinical practice. |
| The Norwegian version of the Self-Efficacy in Clinical Performance Scale (SECP) | This electronic questionnaire measures the level of your coping beliefs. This is done by making a self-assessment of various claims related to your mastery beliefs. It takes 15 minutes to fill in the questionnaire. | Before the start of the clinical practice period.  After completing the clinical practice period 3 and 9 months after the last day after you finished your clinical practice. |
| The Norwegian version of the Clinical Learning Environment and Supervision Scale (CLES) | This electronic questionnaire measures the level of your satisfaction with the practice environment and the guidance. This is done by making a self-assessment of various claims related to your satisfaction with the practice environment and the guidance. It takes 15 minutes to fill in the questionnaire. | After completing the internship period. |
| The Norwegian version of the Technology Acceptance Model (TAM). | This questionnaire measures the extent to which you accept new technology. This is done by making a self-assessment of various claims related to the use of new technology. It takes 15 minutes to fill in the questionnaire. | After training in the new guidance model and after completing the clinical practice period. |
| Evaluation of the feasibility study (feasibility study) | This questionnaire measures your experiences with participation in a feasibility study. It takes 15 minutes to fill in the questionnaire. | After completion of feasibility study (feasibility study) |

**Participation is voluntary**

Participation in the project is voluntary. You can withdraw your consent for participation in the project at any time, right up until the final results have been published in a scientific journal. You will then have no further obligations in relation to participation in the project. Regardless of when you withdraw your consent, the data collected about you will be deleted until then. For example, if you withdraw your consent after you have answered the questionnaires, you must notify us by email that you wish to withdraw your consent for participation. We will then delete your results from answering the questionnaire. We will also delete the information you have provided when registering for the project (name, email address, gender, age, previous education and previous experience from the healthcare system). To withdraw your consent, you must contact Jaroslav Zlamal (Jaroslav.Zlamal@ldh.no).

**Your privacy - more about the use of your personal data**

We will only use the information about you for the purposes we have described in this article. We treat the information confidentially and in accordance with the privacy regulations.

After you have registered for the project (completed form "Registration for the project"), we will download your personal data to a password-protected USB key. We will also collect your name, email address, gender, age, previous education and previous experience from the healthcare system under a code, a connection key. Only this connection key will identify you in the project. Only the PhD candidate Jaroslav Zlamal and associate professor Andrea Aparecida Goncalves Nes will have access to these connection keys.

The supplier of the Norwegian version of the Health Sciences Reasoning Test (HSRT) is Insight Assessment. Insight Assessment will analyze collected data from the critical thinking test. When you are asked to complete this test, you must enter a username and password that you will receive in advance from the PhD candidate. Your username will be collected under the connection key together with other information, as described above.

Insight Assessment only receives your answers, i.e. does not have access to your name, email, gender or other information that you have provided when registering. This part of the data collection is carried out anonymously.

The questionnaires NCS, SECP, CLES, TAM and "Evaluation of feasibility study" will be answered through Questback.

When you fill in these questionnaires, you will have to enter your email address. After you have submitted your answers, your email address will be deleted and replaced by a link key.

You will not receive results from the completion of your critical thinking test (HSRT), NCS, SECP, CLES, TAM or "Feasibility study evaluation".

If you do not answer the questionnaires within the set deadline, you will only receive up to 2 reminders by email with an invitation to fill in the questionnaire.

The recorded audio file from the focus group interview is transcribed and the transcription is saved on an encrypted and password protected USB key.

Results from the tests/questionnaires are published in scientific journals. The participants will not be recognizable in the published articles.

**What happens to your information when we end the research project?**

Expected project end is approx. April 2023. Name, email address, gender, age, previous education and work experience that you provided when registering for the project will be deleted after the end of the project.

**Your rights**

As long as you can be identified in the data material, you have the right to:

access to which personal data is registered about you, and to have handed over

a copy of the information,

to have personal data about you corrected,

to have personal data about you deleted, and

to send a complaint to the Norwegian Data Protection Authority about the processing of your personal data

Participation in the project in no way affects the assessment of you or your academic progress in your education. Withdrawal of your consent has no negative consequences for you. Researchers participating in the project are not involved and will not be involved in teaching or practical follow-up of participants while the project is taking place.

**What gives us the right to process personal data about you?**

We process information about you based on your consent.

Commissioned by Lovisenberg Diaconal University College, NSD – Norwegian Centre for Research Data AS has assessed that the processing of personal data in this project is in accordance with the privacy regulations.

**Where can I find out more?**

**If you have questions about the study, or wish to make use of your rights, please contact:**

*Lovisenberg Diaconal University College* - *Jaroslav Zlamal. Contact:*

[*Jaroslav.Zlamal@ldh.no*](mailto:Jaroslav.Zlamal@ldh.no)

*Lovisenberg Diaconal University College - Andrea Aparecida Goncalves Nes. Contact:* [*andrea.nes@ldh.no*](mailto:andrea.nes@ldh.no)

*Our data protection officer: Eirik Meisinget Johansen. Contact:* [*eirik.meisingset.johansen@ldh.no*](mailto:eirik.meisingset.johansen@ldh.no)

If you have questions related to NSD's assessment of the project, you can contact:

NSD – Norwegian Centre for Research Data AS email: ([personverntjenester@nsd.no](mailto:personverntjenester@nsd.no)) or telephone number: 55 58 21 17.

Best regards,

*Responsible for the project*

(Andréa Aparecida Goncalves Nes)

-------------------------------------------------------------------------------------------------------------------------

**Declaration of consent**

*I have received and understood information about the project Technology-supported guidance model to stimulate nursing students' critical thinking and competence. I agree to:*

to participate in the research project as described above

that my information is processed until the project is finished

Submission of the registration form in Questback is considered consent to participation.
