# Supplementary material for: A Technology-Supported Guidance Model to Support the Development of Critical Thinking Among Undergraduate Nursing Students in Clinical Practice: Concurrent, Exploratory, Flexible, and Multimethod Feasibility Study
Source: JMIR Form Res. 2023 Apr 26;7:e43300. doi: 10.2196/43300 (PMC10173047; doi:10.2196/43300)
Supplement: Multimedia Appendix 5 [file formative_v7i1e43300_app5.docx]

**Multimedia Appendix 5.** Overview of quantitative data not included in the study.

Results regarding application usage, digital assessment, daily reports and feedback.

| Perceived experience | TOPP-N Application Usage | | | Digital assessment | | | Receiving and giving feedback | | Fulfilling of daily reports | |
| --- | --- | --- | --- | --- | --- | --- | --- | --- | --- | --- |
|  | NS N=4 | NP N=3 | NE N=1 | NS N=4 | NP N=3 | NE N=1 | NS N=4 | NP N=3 | NS N=4 | NP N=3 |
| Time consuming | 3 | 3 | - | 3 | 3 | 0 | 3 | 3 | 3 | 2 |
| Frustrating | 3 | 3 | - | - | - | - | - | - | - | - |
| Boring | 3 | 3 | - | - | - | - | - | - | - | - |
| Stressful | 3 | 3 | - | - | - | - | - | - | 3 | 2 |
| Disturbing | 3 | - | - | - | - | - | - | - | - | - |
| Inspirational | 1 | 3 | 1 | - | - | - | - | - | - | - |
| Exciting | 1 | - | 1 | - | - | - | - | - | - | - |
| Meaningful | 1 | 1 | 1 | 3 | 3 | 1 | - | - | - | - |
| Easy | - | 3 | - | - | - | - | - | - | - | - |
| Exhausting | - | - | - | - | - | - | 3 | 3 | 3 | - |
| Positive | - | - | - | - | - | 1 | 3 | 3 | - | - |
| Useful | - | - | - | 3 | 3 | 1 | 3 | 3 | - | - |
| Informative | - | - | - | - | - | - | 3 | 3 | - | - |

NS=nursing students

NP=nurse preceptors

NE=nurse educators

(-)=not answered

Participants perceived experience and intention to use TOPP-N further.

| Perceived experience | | Future use of the TOPP-N | | | |
| --- | --- | --- | --- | --- | --- |
|  | | NS (N=4) | NP  (N=3) | NE  (N=1) | |
| Prefer not to use it in the future | | 2 | 0 | 0 | |
| Want to use it in the future | | 2 | 3 | 1 | |
| Users gained insight in what is expected from them in the clinical practise | | 3 | 3 | 1 | |
| TOPP-N application has contributed to better communication between users | | 2 | 1 | 1 | |
| TOPP-N application has not contributed to better communication between users | | 2 | 2 | 0 | |
| Users have received sufficient information before the start of the feasibility study. | | 2 | 3 |  | |
| Users have received sufficient training on the TOPP-N application before the start of the feasibility study | | 4 | 1 | 1 | |
| Reported time usage of TOPP-N | | | | | |
| Time usage of the TOPP-N app | | | | | |
|  | NS (N=4)* | NP (N=3)** | | | NE (N=1) |
| Fulfilling of daily reports | 20 - 45 min | 30 - 120 min | | | N/A |
| Mid- term assessment | 45 - 120 min | 45 - 90 min | | | 34 - 60 min |
| Final-term assessment | 120 - 240 min | 30 - 60 min | | | 30 min |

**Two of the nurse preceptors did not use the TOPP-N application daily.

*one of the participants did not answer the time usage for mid- and final term assessment
